# Supplementary material for: Discovery and application of insertion-deletion (INDEL) polymorphisms for QTL mapping of early life-history traits in Atlantic salmon
Source: BMC Genomics. 2010 Mar 8;11:156. doi: 10.1186/1471-2164-11-156 (PMC2838853; doi:10.1186/1471-2164-11-156)
Supplement: Additional file 2 — Information on developed 76 locus single-run INDEL panel in Atlantic salmon. Information on fluorescence labeling, primer concentrations, PCR pooling and links to alignments, INDEL motifs and GENESCAN (Burge and Karlin 1997) predictions of genes/exons are available in html format. [file 1471-2164-11-156-S2.ZIP › Additionalfile2/snpsummary7157.html]

```
Cluster 2054 Contig 1

prev  Summary    Contig List  next
```

Size of Consensus sequence = 1356

Number of sequences = 26

Minimum redundancy = 6

Key

A gi|45322544|gb|CK892811.1|CK892811 SGP152877 Atlantic salmon Skin cDNA library Salmo salar cDNA clone HU5-0443 5', mRNA sequence  
B gi|117550430|gb|EG881722.1|EG881722 EST\_ssal\_eve\_24911 ssaleve thyroid Salmo salar cDNA Salmo salar cDNA clone ssal\_eve\_533\_351\_rev 5', mRNA sequence  
C gi|89829263|gb|DY692030.1|DY692030 EST\_ssal\_plnb\_4940 ssalplnb mixed\_tissue Salmo salar cDNA Salmo salar cDNA clone ssal\_plnb\_025\_104\_rev 5', mRNA sequence  
D gi|117510827|gb|EG842586.1|EG842586 EST\_ssal\_eve\_9781 ssaleve thyroid Salmo salar cDNA Salmo salar cDNA clone ssal\_eve\_513\_091\_rev 5', mRNA sequence  
E gi|89875783|gb|DY731906.1|DY731906 EST\_ssal\_rgb2\_87645 ssalrgb2 mixed\_tissue Salmo salar cDNA Salmo salar cDNA clone ssal\_rgb2\_643\_143\_fwd 3', mRNA sequence  
F gi|24389032|gb|CA058789.1|CA058789 ssalrga506191 mixed\_tissue Salmo salar cDNA, mRNA sequence  
G gi|29321777|gb|CB510551.1|CB510551 ssalnwh506186 whole Salmo salar cDNA, mRNA sequence  
H gi|24387911|gb|CA057668.1|CA057668 ssalrgb507078 mixed\_tissue Salmo salar cDNA, mRNA sequence  
I gi|24383070|gb|CA052827.1|CA052827 ssalrga507334 mixed\_tissue Salmo salar cDNA, mRNA sequence  
J gi|24393227|gb|CA062984.1|CA062984 ssalrgb524309 mixed\_tissue Salmo salar cDNA, mRNA sequence  
K gi|117504655|gb|EG836414.1|EG836414 EST\_ssal\_eve\_11063 ssaleve thyroid Salmo salar cDNA Salmo salar cDNA clone ssal\_eve\_515\_010\_rev 5', mRNA sequence  
L gi|89865532|gb|DY721655.1|DY721655 EST\_ssal\_rgb2\_77394 ssalrgb2 mixed\_tissue Salmo salar cDNA Salmo salar cDNA clone ssal\_rgb2\_627\_020\_fwd 3', mRNA sequence  
M gi|29326155|gb|CB514929.1|CB514929 ssalrgb553032 mixed\_tissue Salmo salar cDNA, mRNA sequence  
N gi|89829264|gb|DY692031.1|DY692031 EST\_ssal\_plnb\_4941 ssalplnb mixed\_tissue Salmo salar cDNA Salmo salar cDNA clone ssal\_plnb\_025\_104\_fwd 3', mRNA sequence  
O gi|57125949|gb|CX357390.1|CX357390 ssalrgb524309\_rev\_0 mixed\_tissue Salmo salar cDNA, mRNA sequence  
P gi|89854252|gb|DY710375.1|DY710375 EST\_ssal\_rgb2\_66114 ssalrgb2 mixed\_tissue Salmo salar cDNA Salmo salar cDNA clone ssal\_rgb2\_607\_215\_rev 5', mRNA sequence  
Q gi|57123395|gb|CX354836.1|CX354836 ssalrgb553032\_rev\_0 mixed\_tissue Salmo salar cDNA, mRNA sequence  
R gi|117510838|gb|EG842597.1|EG842597 EST\_ssal\_eve\_9782 ssaleve thyroid Salmo salar cDNA Salmo salar cDNA clone ssal\_eve\_513\_091\_fwd 3', mRNA sequence  
S gi|89875784|gb|DY731907.1|DY731907 EST\_ssal\_rgb2\_87646 ssalrgb2 mixed\_tissue Salmo salar cDNA Salmo salar cDNA clone ssal\_rgb2\_643\_143\_rev 5', mRNA sequence  
T gi|85025711|gb|DW554367.1|DW554367 EST\_ssal\_rgb2\_18786 rgb2 Salmo salar cDNA clone ssal\_rgb2\_530\_281\_rev 5', mRNA sequence  
U gi|89845551|gb|DY701674.1|DY701674 EST\_ssal\_rgb2\_57413 ssalrgb2 mixed\_tissue Salmo salar cDNA Salmo salar cDNA clone ssal\_rgb2\_592\_257\_rev 5', mRNA sequence  
V gi|89860446|gb|DY716569.1|DY716569 EST\_ssal\_rgb2\_72308 ssalrgb2 mixed\_tissue Salmo salar cDNA Salmo salar cDNA clone ssal\_rgb2\_617\_058\_rev 5', mRNA sequence  
W gi|117504656|gb|EG836415.1|EG836415 EST\_ssal\_eve\_11064 ssaleve thyroid Salmo salar cDNA Salmo salar cDNA clone ssal\_eve\_515\_010\_fwd 3', mRNA sequence  
X gi|117550429|gb|EG881721.1|EG881721 EST\_ssal\_eve\_24910 ssaleve thyroid Salmo salar cDNA Salmo salar cDNA clone ssal\_eve\_533\_351\_fwd 3', mRNA sequence  
Y gi|89859657|gb|DY715780.1|DY715780 EST\_ssal\_rgb2\_71520 ssalrgb2 mixed\_tissue Salmo salar cDNA Salmo salar cDNA clone ssal\_rgb2\_615\_348\_rev 5', mRNA sequence  
Z gi|89865531|gb|DY721654.1|DY721654 EST\_ssal\_rgb2\_77393 ssalrgb2 mixed\_tissue Salmo salar cDNA Salmo salar cDNA clone ssal\_rgb2\_627\_020\_rev 5', mRNA sequence

3 SNPs detected

A B C D E F G H I J K L M N O P Q R S T U V W X Y Z  cosegregation weighted

251 A - - A - - - - - A A A A - . . . . . . . . . . . .   2/3 35.90
252 C - - C - - - - - C C C C - . . . . . . . . . . . .   2/3 35.90
730 . . . . . . . . . . C T T C T T . . C T C T C . C T   1/3 16.67
